# Mimicking the myoseptum in cultivated fish by manufacturing edible microalgae-rich nanofibers

**DOI:** 10.1038/s41538-025-00508-6

**Published:** 2025-07-21

**Authors:** Diana M. C. Marques, Bernardo D. Pereira, Beatriz Malhão, João C. Silva, Paola Sanjuan-Alberte, Frederico Castelo Ferreira

**Affiliations:** 1 Department of Bioengineering and Institute for Bioengineering and Biosciences, Instituto Superior Técnico, Universidade de Lisboa, Lisbon, Portugal; 2 Associate Laboratory i4HB—Institute for Health and Bioeconomy, Instituto Superior Técnico, Universidade de Lisboa, Lisbon, Portugal; 3 Department of Mechanical and Aerospace Engineering and PolitoBIOMed Lab, Politecnico di Torino, Turin, Italy

**Keywords:** Bioinspired materials, Marine biology

## Abstract

Cultivated fish can offer seafood with lower environmental impact and improved animal welfare. Electrospun fibres, resembling collagen in structure and size, can mimic the myoseptum —an important structural element for fish fillet patterning. Here, we cultured *Dicentrarchus labrax* Embryonic Cells (DLEC) for the first time on edible zein-gelatin electrospun fibres. Notably, we successfully incorporated biomass of the microalgae *Nannochloropsis oceanica* into the fibres. The effect of the Maillard reaction as thermal crosslinking method was studied. The structure, chemical composition, mechanical performance and biocompatibility of the electrospun fibres were evaluated. Fibres containing *N. oceanica* biomass significantly enhanced DLEC adhesion, leading to faster proliferation. Aligned fibres promoted DLEC alignment, an important feature for improving texture in food products. Finally, the fibrous scaffolds and edible bioinks were successfully combined to fabricate a cultivated fish prototype. Overall, these findings highlight the potential of edible electrospun fibres for the development of cultivated fish fillets.

## Introduction

Cultivated/cultured meat is a promising manufacturing strategy to provide alternative food products to the consumers, potentially addressing several environmental and ethical concerns associated to conventional meat production^1,2^. Cultured meat is based on tissue engineering technologies and involves a cell isolation step (biopsy), followed by cell expansion and differentiation in bioreactors to generate large quantities of cell mass from specific tissues, such as muscle^3^. Afterwards, to assemble and create the structure of meat, cells are usually combined with an edible scaffold. Electrospun fibres have the potential to function as scaffolds to support muscle cell growth and differentiation due to their fibrous and muscle-like intrinsic morphology^4,5^.

Cultured fish production follows the same rationale but presents a few additional challenges, particularly the limited availability of fish cell lines and specific antibodies for their characterization—the latter being a major bottleneck for characterizing the obtained cells and tissues. Nevertheless, Saad et al. generated an immortalized Atlantic mackerel muscle cell line, enabling the study of fish muscle cell growth and differentiation^6^. Tsuruwaka and Shimada also demonstrated that is possible to derive fish cells with differentiation capacity from fish fins^7^ and, more recently, Xu et al. isolated fish satellite cells from a large yellow croaker and combined them with fish adipose-derived stem cells to create a structured fish product^8^.

Herein, we propose a novel strategy to manufacture edible electrospun fibres that allow fish cell adhesion and growth to be applied in the production of cultured fish. Electrospun nanofibres have great potential as scaffolds in cultured fish as they can closely resemble the myoseptum of fish fillets. The myoseptum is the connective tissue that allows for the fish fillet patterning, as it separates the myomeres^9^. Besides its importance in the visual appearance of the fish fillet, the myoseptum also contributes to the mechanical integrity and stability of the tissue, and consequently to its texture.

To the best of our knowledge, there are no studies on electrospun nanofibres as scaffolds for fish cells. Nonetheless, this technology has recently been explored for cultured meat. In 2023, Santos et al. showed that cellulose acetate fibres loaded with annatto extract, isolated from the fruit annatto (*Bixa orellanna L*.), can support C2C12 skeletal muscle cells proliferation^10^. In the same year, Melzener et al. demonstrated that short-stranded zein fibres combined with RGD-functionalized alginate enhance bovine muscle precursor cell alignment^11^. In 2024, Santos et. al showed that besides C2C12, cellulose acetate fibres also support the growth and differentiation of chicken H9c2 myoblasts^12^. While cellulose acetate is not easily digestible by humans, the authors demonstrated that stacking the electrospun constructs makes it possible to produce cultivated chicken products with sufficient mechanical properties to be fried at 200 °C.

Scaffolds such as electrospun fibres could be a platform to grow large cell numbers for cultured meat and fish production. These scaffolds may function solely as supports for cell growth and differentiation or be incorporated into the final product. Therefore, there are certain requirements that electrospun fibres should present when used for cultured meat and fish applications. Electrospun fibres should be non-harmful for human health, digestible, and edible —features typically not found in fibres designed for tissue engineering purposes. Importantly, these fibres must avoid the use of animal-derived components and be fabricated using food-grade and non-toxic solvents such as ethanol and acetic acid^13,14^. In the aforementioned studies^10–12^, the fibres were electrospun from solutions of cellulose acetate dissolved in mixtures of acetone and N, N-dimethylformamide (DMF), while the zein fibres were dissolved in mixtures of acetic acid and ethanol. Although DMF is highly effective for electrospinning, it is considered toxic to human health. Therefore, in this work, we produced edible electrospun fibres composed of zein, gelatine, and glucose, using exclusively ethanol or acetic acid as solvents.

The scaffolds incorporated into cultured meat products present also the opportunity to enrich the final product with relevant nutritional and organoleptic qualities. In cultured fish production, in addition to support fish cell growth, scaffolds can also deliver flavouring agents, umami taste and odour-active seafood compounds^15^. Different sources of microalgae with high omega-3 long-chain polyunsaturated fatty acids (PUFAs) content have been previously described as organoleptic enhancers^16,17^. *Nannochloropsis oceanica* (NO) is an example of such microalgae and was already used as a food ingredient^18^. In 2018, Rodríguez De Marco et al. showed that the incorporation of *N. oceanica* in food products enhances consumer acceptance due to its fishy flavour^19^, provided by the presence of different volatile organic compounds (VOCs)^20^. Moreover, we previously demonstrated that adding *N. oceanica* to printable inks enhances their smell and taste^21^. We hypothesize that *N. oceanica* biomass could have an impact on the electrospun fibres organoleptic properties produced for cultured fish applications. Therefore, in this work we also explored the addition of NO into electrospun fibres.

To summarize, this study investigates the potential of electrospun zein-gelatine fibres produced using ethanol or acetic acid, as solvents, to sustain fish cell adhesion and proliferation. For that, we studied the morphological, molecular, and mechanical characteristics of the fibres after crosslinking with glucose and incorporation of *N. oceanica* biomass. Furthermore, this work introduces a novel conceptual approach to integrating electrospun fibres into cultured fish fillets. Overall, we demonstrate that electrospinning is a reliable technique to produce edible electrospun scaffolds with enhanced organoleptic properties, which can support fish cell culture and to mimic the native fish myoseptum.

## Results

### Impact of solvent and composition on the zein-based fibres morphology

**Fig. 1:**
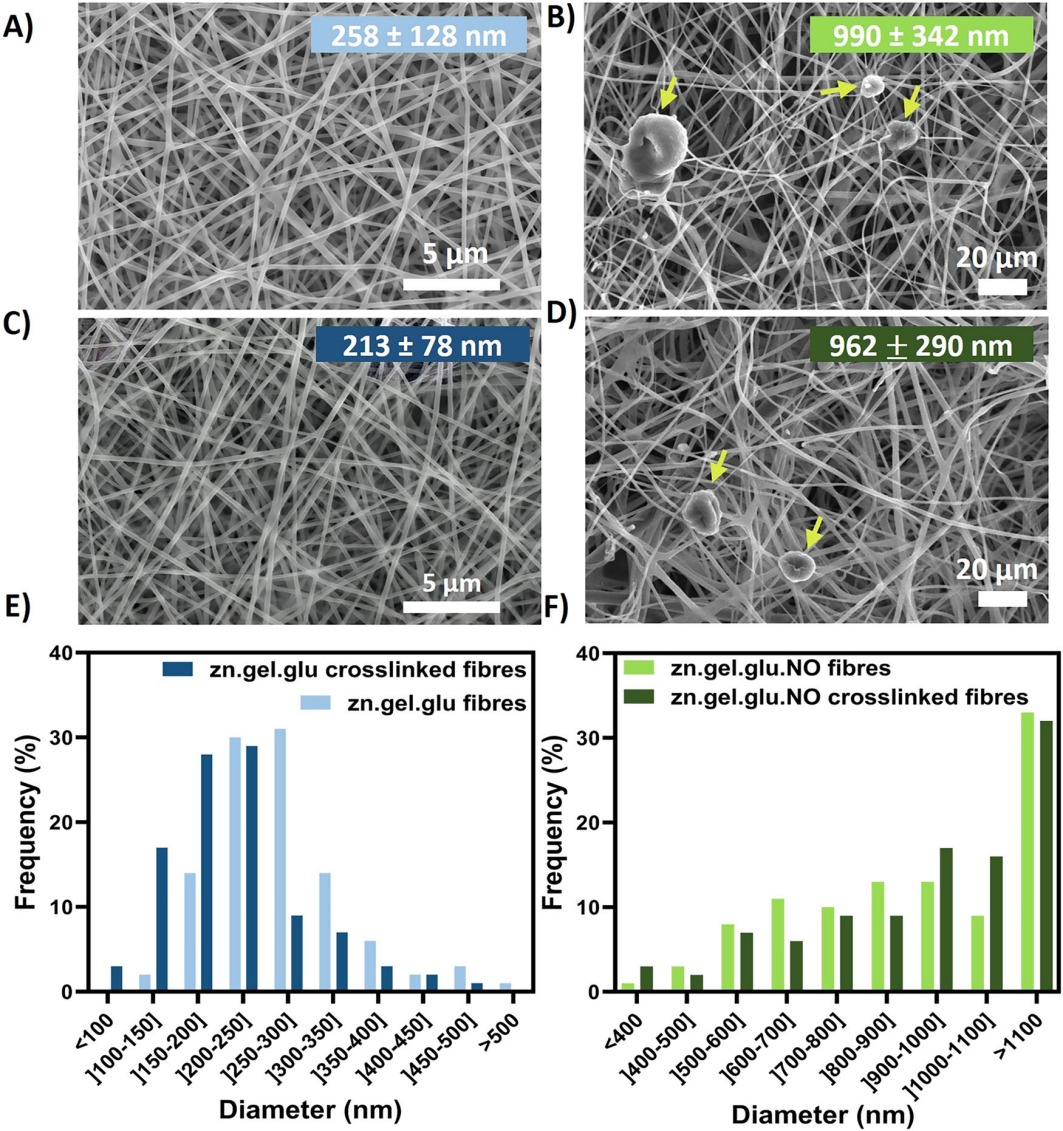
SEM analyses of zn.gel.glu fibres and zn.gel.glu.NO fibres. SEM micrographs. **A** zn.gel.glu fibres, **B** zn.gel.glu.NO fibres, **C** zn.gel.glu crosslinked fibres, and (**D**) zn.gel.glu.NO crosslinked fibres, as well as the respective fibre diameter distribution histograms (**E**, **F**). The yellow arrows are pointing to *N. oceanica* (NO). The average fibre diameter values for each condition are represented as mean ± SD and are depicted in the respective image. Scale bar: 5 μm (**A**, **C**); 20 μm (**B**, **D**).

Zein electrospun nanofibres were initially fabricated to evaluate the impact of different food-grade solvents and zein concentrations in a homemade electrospinning set-up, after optimizing the electrospinning parameters. Supplementary Fig. S1 shows their morphology when assessed by SEM. The fibrous scaffolds produced with ethanol exhibited average diameters within the range of 390–800 nm, while the fibres fabricated using acetic acid show diameters ranging from 312 to 416 nm. It was also observed an increase in fibre diameter with increased zein concentrations, specifically, when using ethanol. Fibres combing zein, gelatine and glucose (zn.gel.glu) and fibres containing zein, gelatine, glucose and *N. oceanica* (zn.gel.glu.NO) were successfully fabricated using acetic acid. SEM micrographs exhibiting the fibres morphology, with and without the crosslink reaction can be seen in Fig. 1. The average diameters of the zn.gel.glu fibres before and after crosslinking consisted of 258 ± 128 nm and 213 ± 78 nm, respectively. These fibres were smaller than the zn.gel.glu.NO fibres, which presented values before and after crosslinking of 990 ± 342 nm to 962 ± 290 nm, respectively. Thus, the addition *of N. oceanica* biomass significantly increased the electrospun fibres average diameters. On the other hand, the thermal crosslink reaction (Maillard reaction) that occurs at 140 °C led to a decrease in fibre diameters as previously reported in the literature^22^. Despite this, we also observed that the fibrous scaffolds presented no significant structural differences before and after the Maillard crosslinking reaction, which is also in accordance with the literature^23^. Bashur et al. showed that for PLGA electrospun fibres with diameter of 140 nm, 780 nm, and 3600 nm resulted in equal NIH 3T3 fibroblasts adherence efficiency^24^. In addition to having large fibres on average, the produced fibres also contain 55–60% nanofibres with sizes ranging from 400 to 1000 nm. We hypothesise that this variation in diameter may increase the number of focal adhesion sites available for cell attachment, potentially making all the produced fibrous scaffolds appropriate for fostering cell growth.

### Chemical composition of the zn.gel.glu and zn.gel.glu.NO fibres

**Fig. 2:**
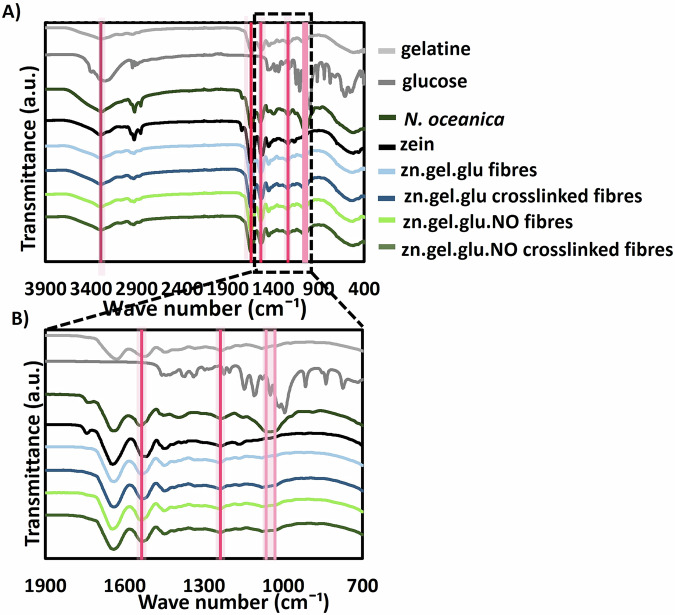
FTIR analysis of zn.gel.glu fibres, zn.gel.glu crosslinked fibres, zn.gel.glu.NO fibres, and zn.gel.glu.NO crosslinked fibres and the respective zn, gel, glu, and NO individual materials. (**A**) full FTIR spectrum, and (**B**) amplification of FTIR region between 1300 and 900 cm^-1^.The highlighted dotted vertical lines focus on the characteristic peaks that confirmed several interactions between the electrospun fibres materials with and without crosslinking.

The chemical features of zn.gel.glu and zn.gel.glu.NO fibres with and without crosslink were characterized by FTIR (Fig. 2). All fibrous scaffolds display a common adsorption peak around 3290 cm^−1^, related to the O-H and N-H stretching, due to the hydrogen bonding interactions between gelatine and zein. All electrospun scaffolds also present peaks at 1645 cm^−1^, 1534 cm^−1^ and 1238 cm^−1^ that can be allocated to C=O stretching vibrations of amide I, C-N stretching vibrations of amide II and C-N stretching and N-H bending combination of amide III, respectively. Moreover, the crosslink reaction was confirmed by a reduction of the peaks between 1079 and 1032 cm^−1^, which are associated with the C-O vibrations of glucose, even when adding *N. oceanica* proving that this component does not affect the crosslinking strategy. Finally, no peak splits were observed for the conditions with *N. oceanica*, thus confirming that this component is uniformly dispersed in the fibrous scaffolds.

### Effect of the Maillard reaction and *N. oceanica* incorporation on the mechanical and wettability properties of electrospun nanofibres

**Fig. 3:**
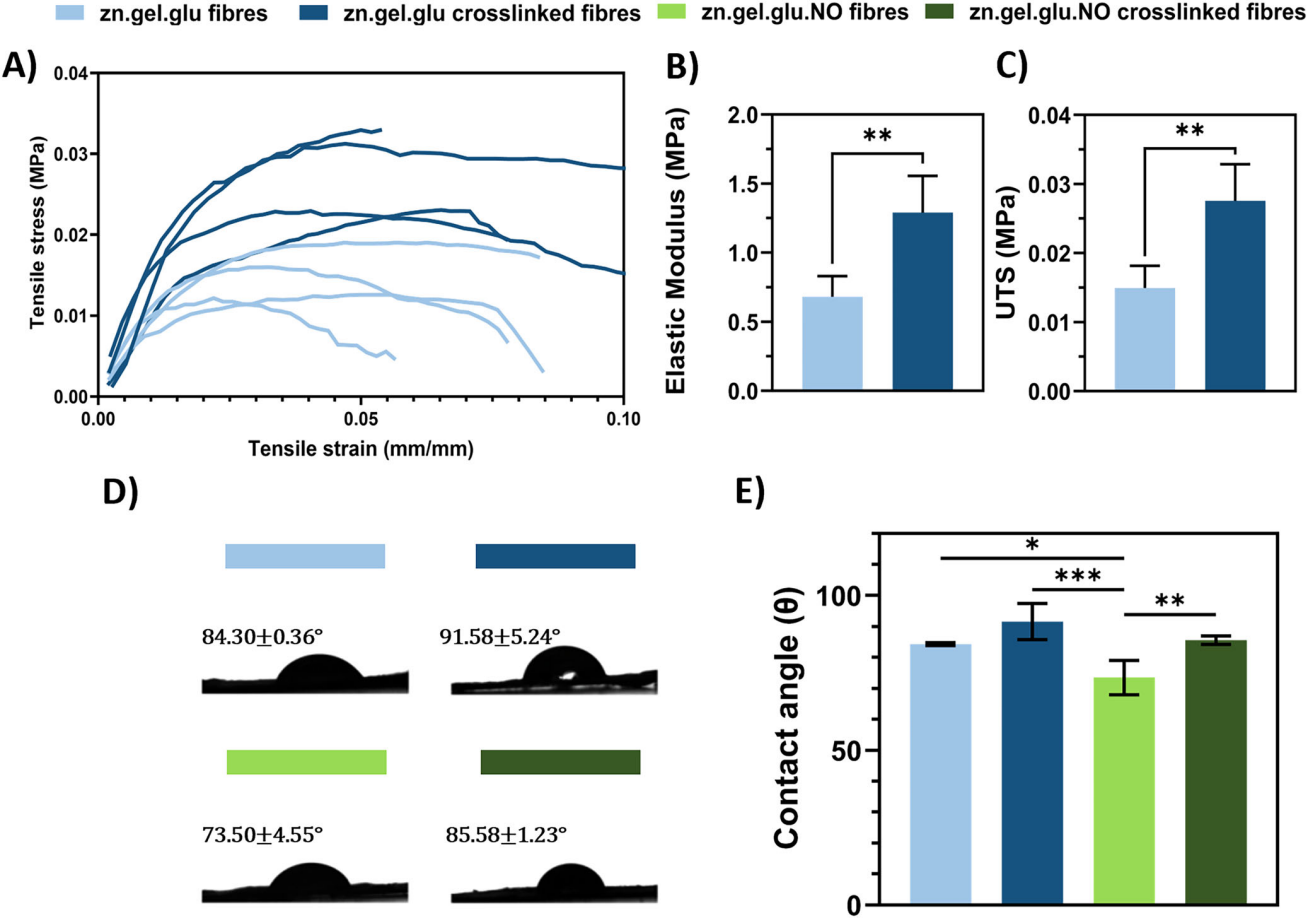
Mechanical and wettability properties of the zn.gel.glu and zn.gel.glu.NO fibrous scaffolds fabricated with and without crosslinking obtained after tensile testing. **A** Stress–strain curves, **B** elastic modulus (**p = 0.0071), **C** ultimate tensile strength (UTS), (**p = 0.0066). Four independent sample specimens (*n* = 4) were used in the analysis. Statistical significance was assessed using the unpaired Student’s *t* test. **D** Sessile drop droplet profile (**E**) and contact angle of the zn.gel.glu and zn.gel.glu.NO fibrous scaffolds fabricated with and without crosslinking. Five different samples (*n* = 5) were used in the analysis. Statistical significance was assessed using ordinary one-way ANOVA and Tukey’s multiple comparison test (ns = *P* value ≥ 0.05; *p = 0.0346; ** = 0.0086; *** = 0.0003).

The mechanical tensile test performed for zn.gel.glu fibres and zn.gel.glu crosslinked fibres is shown in Fig. 3. The different mechanical properties of these two fibres meshes were estimated by the stress–strain curves, shown in Fig. 3A; and quantified by estimating the elastic modulus (Fig. 3B) and the ultimate tensile strength (UTS, Fig. 3C), whose values show that the thermal crosslinking has a statistically significant increase in the mechanical properties of the electrospun fibres. In fact, after crosslinking, the elastic modulus of the fibrous scaffolds increased 1.90-fold (from 0.68 MPa to 1.29 MPa) and the UTS increased 1.8-fold (from 0.015 MPa to 0.027 MPa). Interestingly, the crosslink strategy does not affect the elongation of such fibres, with zn.gel.glu fibres and zn.gel.glu crosslinked fibres having 4.31% and 5.17% elongation at break, respectively (Supplementary Fig. S2). Overall, the crosslinking strategy improved the mechanical properties of the scaffolds, facilitating the handing procedure and avoiding scaffold deformation, while providing mechanical strength similar to those found in fish tissues^25,26^.

The ability of a surface to interact with a liquid, or the wettability of fibrous scaffolds, is a crucial characteristic for cell culture since it is linked to the material’s capacity to support anchorage-dependent cells due to its relationship with cell adhesion and, consequently, with cell growth, migration, and proliferation^27^. Contact angle measurements (Fig. 3D) were performed to estimate the wettability of the zn.gel.glu fibres, zn.gel.glu crosslinked fibres, zn.gel.glu.NO fibres, and zn.gel.glu.NO crosslinked fibres. Figure 3E shows that all scaffolds were slightly hydrophilic (contact angle <90°), except for zn.gel.glu crosslinked fibres (contact angle of 91.58°) that were considered slightly hydrophobic. Interestingly, this increase in hydrophobicity with crosslink reaction was more pronounced for the zn.gel.glu.NO crosslinked fibres, with a 1.16-fold increase in contact angle (from 73.50° to 85.58°), compared to the zn.gel.glu.NO fibres that were not crosslinked.

### Influence of the Maillard reaction and *N. oceanica* incorporation on DLEC adherence and proliferation

**Fig. 4:**
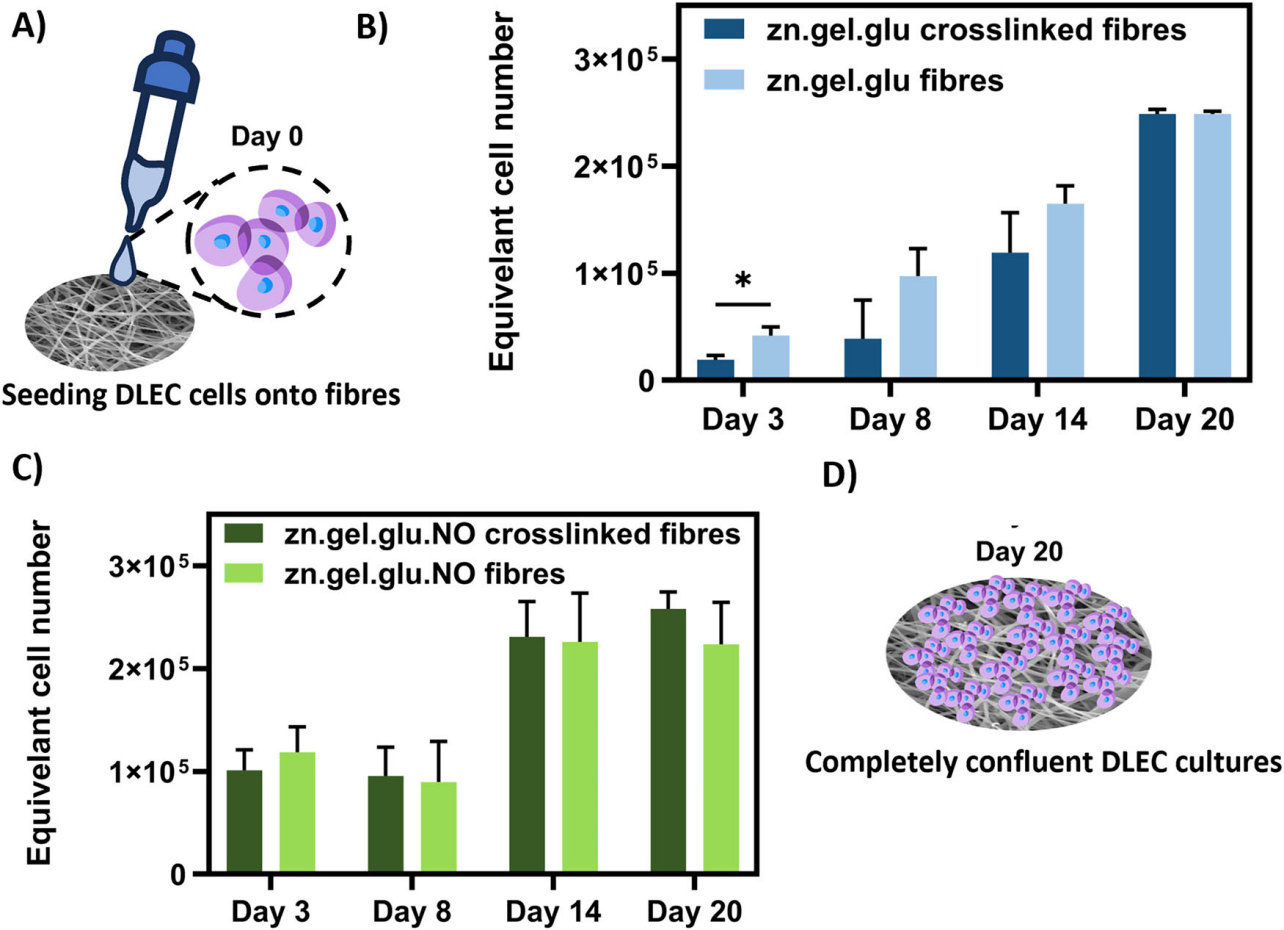
DLEC cultured on randomly oriented fibres meshes. **A** Schematic figure of DLEC cell seeding procedure. AlamarBlue assay results expressed as equivalent numbers of DLEC cells cultured on (**B**) zn.gel.glu fibres and zn.gel.glu crosslinked fibres. **C** zn.gel.glu.NO fibres and zn.gel.glu.NO crosslinked fibres for 20 days. Five different samples (*n* = 5) were used for the analysis. Statistical significance was assessed using ordinary two-way ANOVA and Šidák’s multiple comparison test (ns = *P* value ≥ 0.05; ** = 0.0084; *** = 0.0006). **D** Schematic figure of the fibrous scaffolds after 20 days of culture, hypothesizing completely confluent DLEC cultures.

DLEC cell attachment and proliferation on the electrospun scaffolds throughout a 20-day culture period under static conditions is shown in Fig. 4A. The effect of the Maillard reaction as a crosslinking strategy on DLEC adherence and proliferation was also evaluated, as well as the effect of adding *N. oceanica* to the nanofibres. For that, an AlamarBlue assay was used to monitor DLEC proliferation at days 3, 8, 14 and 20 after seeding. Continuous cell growth on zn.gel.glu and zn.gel.glu crosslinked fibres over the 20-day period is shown in Fig. 4B. Even though we observed significant differences in the equivalent cell number between these conditions on days 8 and 14, on day 20 both conditions reached similar cell numbers. When comparing zn.gel.glu.NO fibres and zn.gel.glu.NO crosslinked fibres (Fig. 4C), we also observed a continuous cell growth during the 20 days of culture. Interestingly, with the addition of *N. oceanica* biomass into the fibres, no statistical differences between the equivalent cell numbers were detected at any time point. Overall, all fabricated fibrous scaffolds supported DLEC cell growth and proliferation. At day 20, high fold increases relative to day 0 were observed in all cases: 4.97 for zn.gel.glu, 4.98 for crosslinked zn.gel.glu, 5.14 for zn.gel.glu and 5.16 for the crosslinked zn.gel.glu.NO fibres, resulting in highly confluent cultures (Fig. 4D) and suggesting a beneficial effect of *N. oceanica* on DLEC cell proliferation.

### Influence of the Maillard reaction and *N. oceanica* incorporation on DLEC morphology

**Fig. 5:**
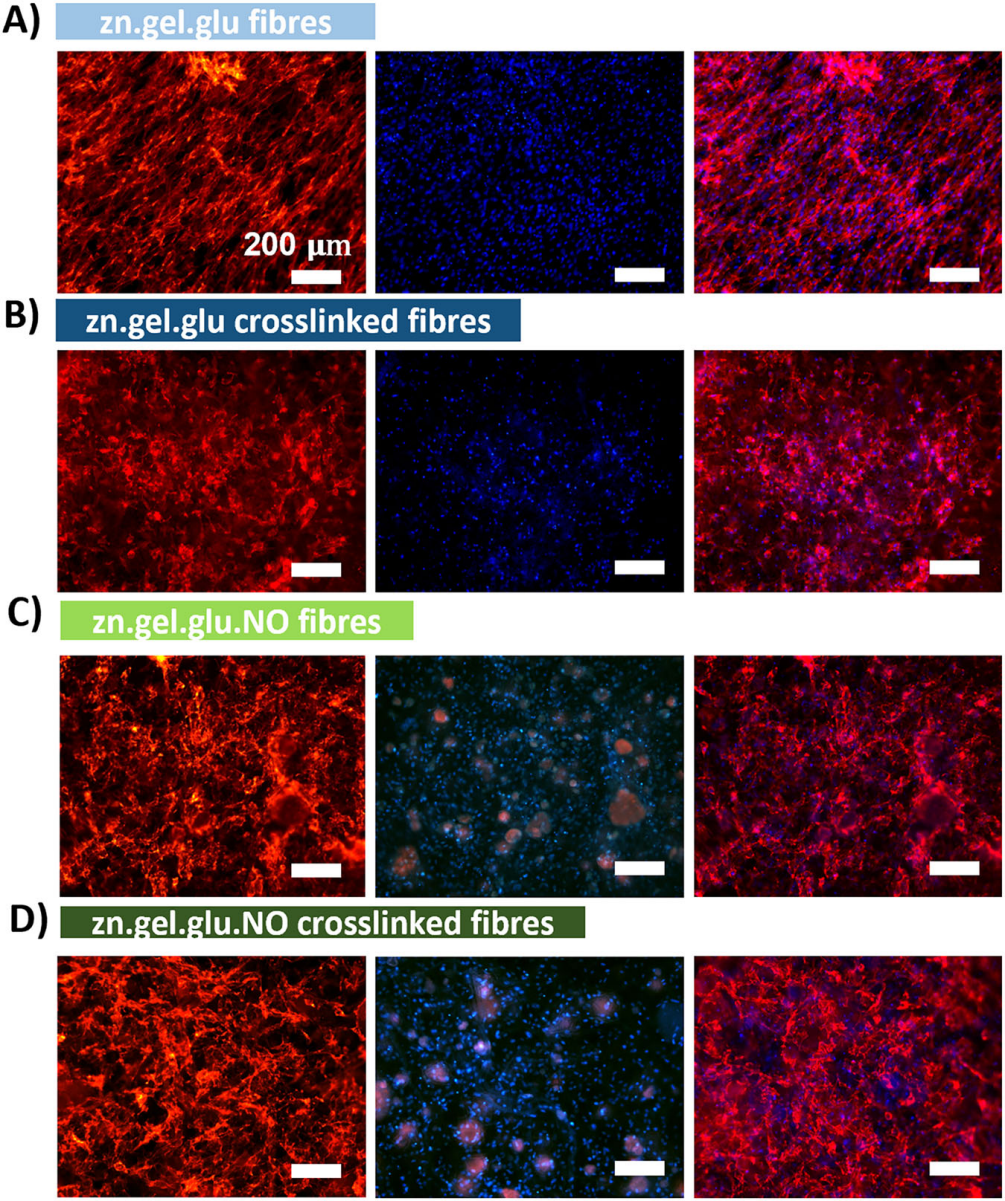
DAPI/Phalloidin staining of DLEC cultured on randomly oriented fibres . Left images: red phalloidin staining of DLEC cytoskeleton; Center images: blue DAPI staining of DLEC nuclei; Right images: DAPI and Phalloidin staining merged images. Staining of DLEC cells cultured on (**A**) zn.gel.glu fibres, **B** zn.gel.glu crosslinked fibres, **C** zn.gel.glu.NO fibres, and (**D**) zn.gel.glu.NO crosslinked fibres, after 20 days of culture. Scale bar: 200 μm.

DAPI/phalloidin staining of DLEC cells was performed after a 20-day culture period to evaluate cell morphology and distribution across the different fibrous scaffolds. From this, we confirmed the high cell density on all scaffolds, validating the successful adhesion and proliferation of DLEC in the fibrous meshes. As shown in Fig. 5, the cells presented a stretched morphology and were spread across the entire surface of all scaffolds. Remarkably, the cells growing on zn.gel.glu fibres (Fig. 5A) were more stretched and aligned in a specific direction, even though the electrospun fibres had a random orientation. In the other conditions (Fig. 5B–D), cells were stretched but mainly aligned in different directions, which is common when culturing cells onto electrospun fibres with random orientations. Lastly, we also confirmed the presence of *N. oceanica* in Fig. 5C, D, by the presence of large red spheres in the DAPI channel corresponding to the microalgae cells.

**Fig. 6:**
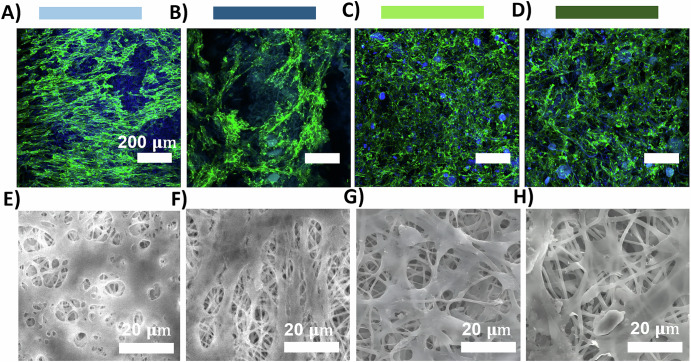
Confocal microscopy images of DAPI/Phalloidin staining using green phalloidin staining for DLEC cytoskeleton and blue DAPI staining for DLEC nuclei. Staining of DLEC cells cultured on (**A**) zn.gel.glu fibres, **B** zn.gel.glu crosslinked fibres, **C** zn.gel.glu.NO fibres, and (**D**) zn.gel.glu.NO crosslinked fibres, after 20 days of culture. Scale bar: 200 μm. SEM micrographs DLEC on top of different fibrous scaffolds after 20 days of culture: **E** zn.gel.glu fibres, **F** zn.gel.glu crosslinked fibres, **G** zn.gel.glu.NO fibres, and **H** zn.gel.glu.NO crosslinked fibres. Scale bar: 20 μm.

Besides growing in the scaffold surface, through confocal microscopy using the DAPI/Phalloidin staining we also observed cells in different layers of the fibrous scaffolds, suggesting that DLEC cells were penetrating onto the scaffold. Curiously, this behaviour was more easily observed for the zn.gel.glu crosslinked fibres (Fig. 6B), zn.gel.glu.NO fibres (Fig. 6C), and zn.gel.glu.NO (Fig. 6D) crosslinked fibres than for cells growing in the zn.gel.glu fibres (Fig. 6A). The respective brightfield images are shown in Supplementary Fig. S3. The DLEC cell morphology was also evaluated through SEM imaging of all fibrous scaffolds (Fig. 6E–H), after 20 days of culture. From these images, we confirmed once again that DLEC cells were adhered to the fibrous scaffolds and proliferated until covering almost the entire surface of such fibrous scaffolds, showing effective cell-to-cell interactions and cell-to-fibre interactions.

### Influence of fibre alignment on DLEC adherence, growth, and morphology

**Fig. 7:**
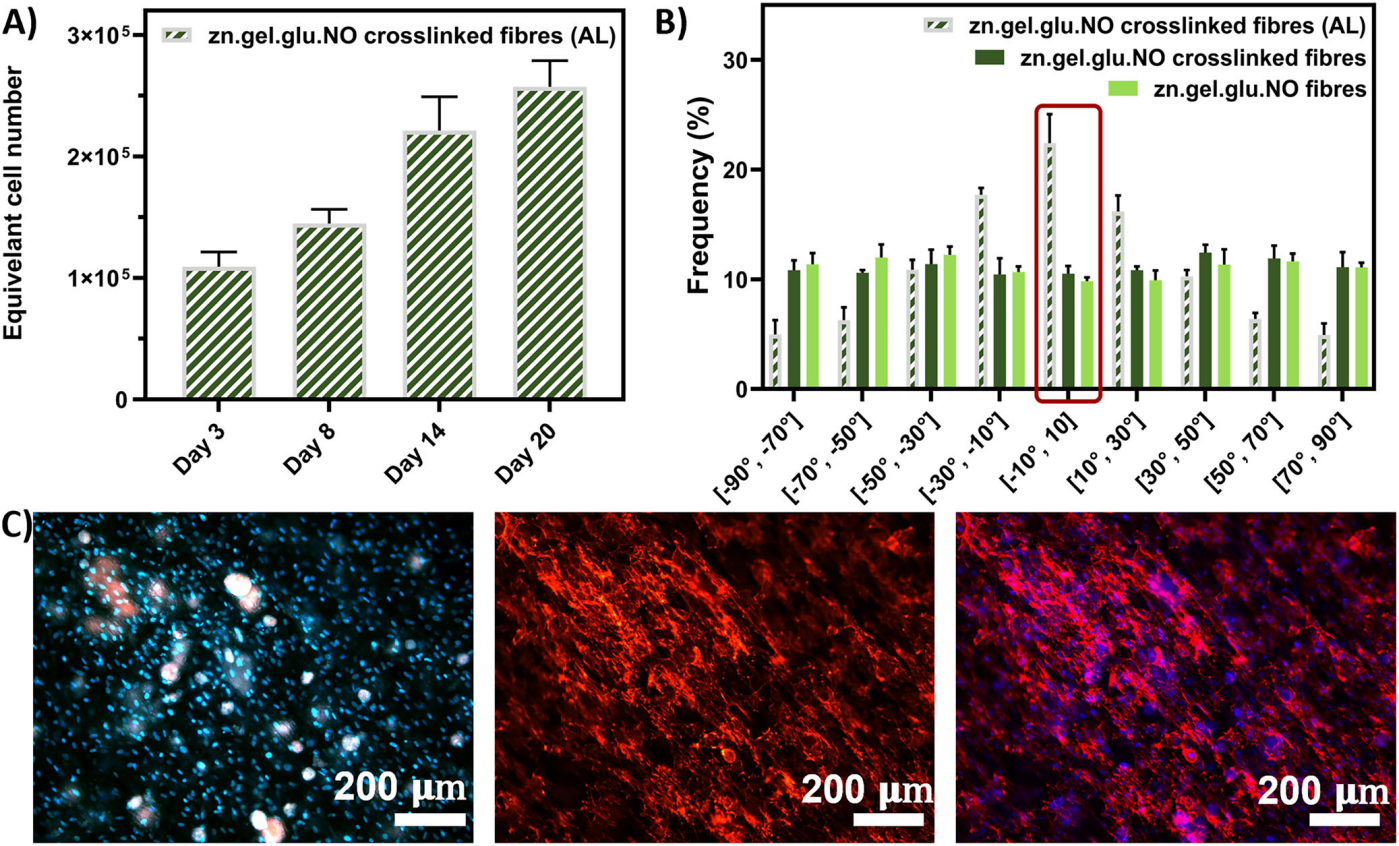
DLEC cultured on aligned fibre meshes and controls. **A** AlamarBlue cell metabolic activity assay of DLEC cultured on zn.gel.glu.NO crosslinked fibres (AL). Five different samples (*n* = 5) were used for the analysis. **B** Distribution of cell orientation throughout the aligned fibrous scaffolds zn.gel.glu.NO crosslinked fibres (AL), zn.gel.glu.NO fibres and zn.gel.glu.NO crosslinked fibres, quantified using Image J (*n* = 3). Statistical significance of the interval [−10°, 10] was assessed using ordinary two-way ANOVA and Šidák’s multiple comparison test (**** = <0.0001). **C** DAPI/Phalloidin staining of DLEC cells cultured on aligned zn.gel.glu.NO crosslinked fibres (AL). Left image: blue DAPI staining of DLEC nuclei; Middle image: red phalloidin staining of DLEC cytoskeleton; Right image: DAPI and Phalloidin staining merged. Scale bar: 200 μm.

The ability of aligned zn.gel.glu.NO crosslinked fibres (zn.gel.glu.NO crosslinked fibres AL), produced with an average diameter of 815 m (Supplementary Fig. S4), to promote DLEC proliferation is shown in Fig. 7A. Similarly to the previous section, we observed continuous cell growth throughout the 20 days with a fold increase (relative to day 0) of 5.15, showing that fibre alignment was not an essential feature to promote cell adherence and proliferation on these fibrous scaffolds in comparison to the random ones (Supplementary Fig. S5). The distribution of orientation (from −90° to 90°) of DLEC cells cultured for 20 days on top of the aligned zn.gel.glu.NO crosslinked fibres (AL) compared to cells that grew on zn.gel.glu.NO fibres and zn.gel.glu.NO crosslinked fibres is presented in Fig. 7B. DLEC cells grown on aligned fibres exhibit a notable peak in the fibre distribution between -10° and 10°, which corresponds to 22.4% of total cell alignment —twice that of the controls with no aligned fibres. In contrast, DLEC cells grown on non-aligned fibres exhibit a uniform distribution across the various degrees of orientation. DAPI/phalloidin staining of DLEC cells after 20 days (Fig. 7C) confirmed the DLEC cell alignment, suggesting that scaffold fibre alignment is an important feature to enhance cell alignment. The respective brightfield image is shown in Supplementary Fig. S6. This study demonstrated that different fibre collection strategies can be used to fabricate the purposed fibrous scaffolds. Moreover, we showed that collecting fibres using parallel plate collectors can modulate fibre alignment, and consequently, cell alignment. As previously mentioned, we hypothesize that such features can contribute to a positive impact on the perceived cultivated fish product texture^28,29^. Finally, we observed cell migration within the fibrous scaffolds (Supplementary Fig. S7), with cells spreading across different scaffold layers.

### Ability of electrospun fibres to recapitulate the native fish myoseptum

**Fig. 8:**
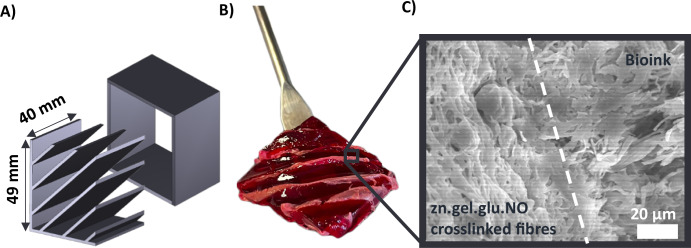
Fish fillet prototype with electrospun fibres meshes mimicking the myoseptum. **A** Model design of a box and a 49 mm × 40 mm insert with six diagonal plates that mimic the fish myoseptum, used to produce (**B**) a fish fillet prototype combining the zn.gel.glu.NO crosslinked fibres (myoseptum) and a 0.9% κ-carrageenan bioink supplemented with beetroot powder. **C** SEM micrograph showing the interface between the zn.gel.glu.NO crosslinked fibres and the bioink. Scale bar: 20 μm.

The ability of the purposed fibrous scaffolds to potentially recapitulate the native fish myoseptum was demonstrated. For this, we designed and fabricated a fish fillet prototype where the myoseptum was mimicked using electrospun fibres. Figure 8A shows the produced prototype composed of a previously reported^30^ edible κ-carrageenan bioink supplement with a food colouring agent that aimed to recapitulate the fish myomere, combined with zn.gel.glu.NO fibres to recapitulate the fish myoseptum. Remarkably, we observed instant adherence of both scaffolds to each other, which was further demonstrated by SEM imaging. Figure 8B highlights the interface between the electrospun fibrous and the bioink where it is possible to observe interactions between the fibrous scaffold and the hydrogel.

## Discussion

Edible electrospun nanofibrous scaffolds are promising platforms to grow cells for cultivated meat and fish applications. Specifically, in cultivated fish fillets, such scaffolds can potentially mimic the myoseptum, a structural element important for fish fillet patterning, visual appearance, mechanical integrity, and fish texture. Therefore, in this study, novel edible microalgae-rich nanofibers were developed and characterized, envisaging their potential use in future cultivated fish applications. We successfully fabricated and characterized novel zn.gel.glu.NO electrospun scaffolds that can potentially mimic the native myoseptum found in conventional fish fillets while also promoting the attachment and proliferation of embryonic-like fish cells.

Initially, we showed that fibre diameters obtained using acetic acid are similar to those of the collagen fibres (10–300 nm) found within the fish myoseptum^31^. For that reason, all scaffolds produced were manufactured using acetic acid. Multiple studies have reported the development of zein-based nanofibres using such solvent. Here, besides zein, we explored for the first time, the use of solutions also containing gelatine, glucose, and *N. oceanica* biomass, a microalga that has already shown to enhance the fish flavour in seafood alternatives. The use of microalgae biomass as part of fibre composition and the implementation of a post-manufacture crosslink reaction (Maillard reaction) were strategies assessed to improve the fibrous scaffolds sensorial and mechanical properties, respectively. The addition of *N. oceanica* biomass significantly increased the electrospun fibres average diameters. Despite having larger fibre diameters than their counterparts without microalgae, zn.gel.glu.NO and zn.gel.glu.NO crosslinked fibres were very good candidates to promote cell adherence and proliferation. We hypothesise, that the diameter heterogeneity of such scaffolds might have contributed to increase the number of focal adherence sites available for cell attachment.

In this study, the mechanical properties of the electrospun scaffolds were also evaluated. We showed that thermal crosslinking (Maillard reaction) increases significantly the elastic modulus (1.90-fold increase) and the UTS (1.8-fold increase) of the scaffolds when compared to the non-crosslinked ones. Besides, the elastic modulus values obtained for the fibrous scaffolds are similar to those reported in the literature for different fish tissues, such as the muscle tissue of the crucian carp (*Carassius auratus*), with reported elastic modulus values of 1.10 MPa^25,26^.

Moreover, the crosslinking strategy had also an impact on the fibre hydrophobicity, leading to higher contact angles. On the other side, the addition of *N. oceanica* into the electrospun scaffolds decreases the fibres contact angle. Wei et al., demonstrated that surfaces with contact angles of 80° are ideal for promoting L929 cell adherence, spreading, and proliferation when compared to super-hydrophobic surfaces (contact angle of 106°)^23^. Therefore, all fabricated fibrous scaffolds are suitable for promoting cell adhesion and growth. However, this indicates that the crosslinking strategy could be responsible for lower cell adherence efficiency, as it increases the scaffold hydrophobicity. In fact, zn.gel.glu crosslinked fibres showed a higher contact angle (91.58°) compared to zn.gel.glu fibres (84.30°). Nonetheless, all fabricated scaffolds successfully promoted fish cell proliferation. Interestingly, *N. oceanica* containing scaffolds with and without crosslinking had no significant differences in cell adherence and proliferation throughout the 20 days of cell culture. We concluded that the microalgae biomass has a beneficial effect on cell adherence and proliferation of the fibres crosslinked using the Maillard reaction. We hypothesize that such effect is related to the lower contact angle of zn.gel.glu.NO crosslinked fibres (85.58°) compared to zn.gel.glu crosslinked fibres (91.58°).

Cell morphology and orientation are other important features for producing cultivated fish, as they are related to improved texture in fish products, such as the fish fillet^30,31^. Here, we show for the first time, that DLEC cells adhere, proliferate (until covering the entire scaffold surface), and penetrate these porous scaffolds by populating their interior. Moreover, when cultured in zn.gel.glu.NO fibrous scaffolds, DLEC cells are able to promote cell-cell and cell-fibre interactions, resulting in stretched and aligned cells. Our findings also demonstrate that different fibre collection strategies can be used to fabricate the proposed fibrous scaffolds with different orientations. As previously mentioned, we hypothesize that such features can contribute positively to the cultivated fish texture^28,29^. Here, we showed that by collecting fibres in parallel collectors is possible to modulate fibre alignment, and consequently, DLEC cell alignment.

Finally, we demonstrated that the electrospun fibres meshes developed in this work, besides serving as a suitable platform for fish cell expansion, are also promising candidates for recapitulating the native fish myoseptum in cultured fish products. For that, we combined the fibrous scaffolds with edible bioinks to fabricate a cultivated fish prototype.

In conclusion, we successfully manufactured, for the first time, edible microalgae-rich nanofibers compatible with seabass cells, capable of mimicking the native fish myoseptum in cultivated fish products such as seabass fillets, while also enhancing its organoleptic properties.

## Methods

### Materials

Zein (zn, MW 22–24 kDa), acetic acid (AA, ≥99.8%, Honeywell), ethanol (EtOH, ≥99.8%, Honeywell), gelatine (gel, type A, gel strength 300, Sigma Aldrich), and glucose (glu) were purchased from Sigma (St. Louis, Missouri USA). *Nannochloropsis oceanica* (NO) powder was purchased from Algikey – Algae Based Solutions S-A (Póvoa de Santa Iria, Portugal).

### Preparation of electrospinning casting solutions

**Fig. 9:**
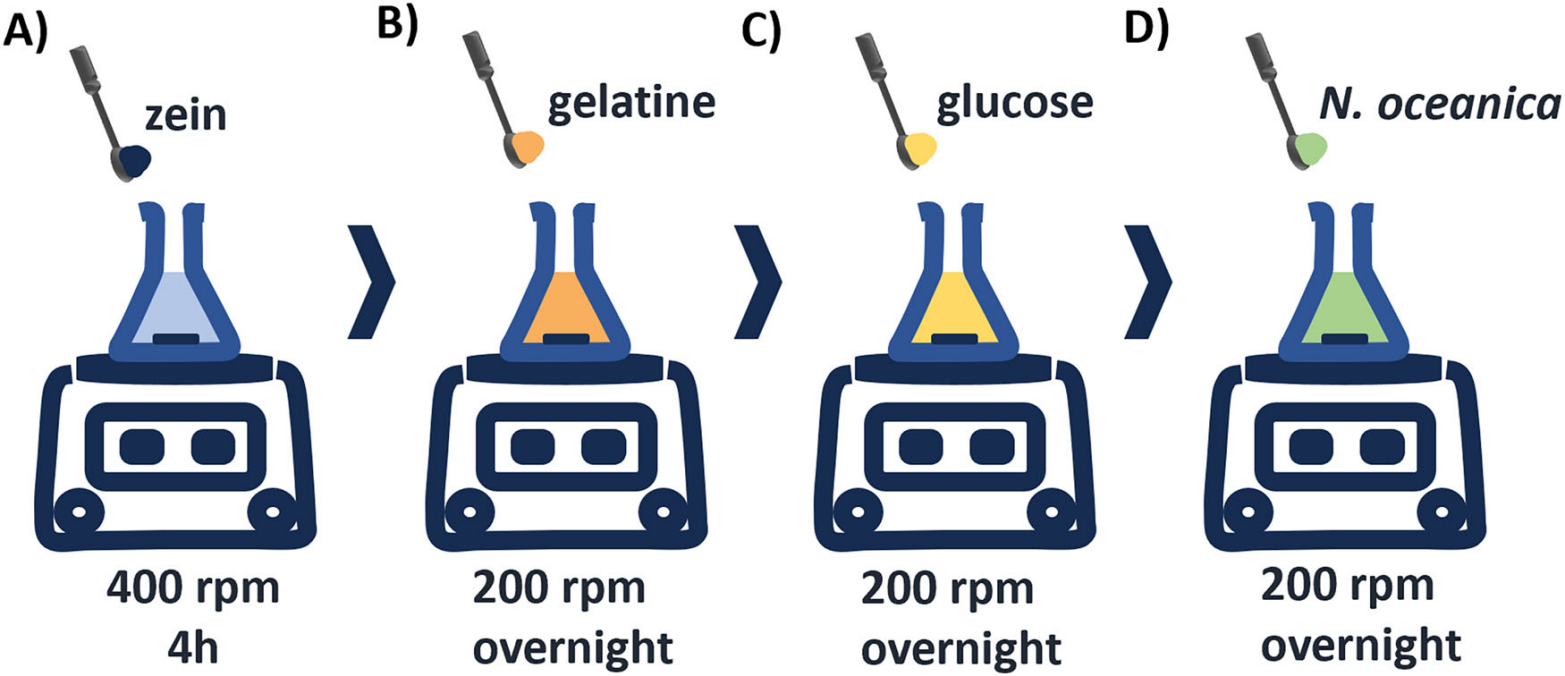
Schematic representation of the electrospinning casting solutions preparation process. Step **A** is required for solutions composed of 30%zein/ethanol, 40%zein/ethanol, 30%zein/acetic acid, 40%zein/acetic acid; Steps (**A**–**C**) are required for the zn.gel.glu fibres, while steps (**A**–**D**) are required for the zn gel.glu.NO fibres. OV overnight.

The preparation of the electrospinning casting solutions is illustrated in Fig. 9. Zein was dissolved at 30% (w/v) and 40% (w/v) in 70% (v/v) ethanol or 80% (v/v) acetic acid and stirred at 400 rpm for 4 h at room temperature (RT) until achieving a homogeneous solution (Fig. 9A). Then, a solution of zein and gelatine was prepared by first dissolving zein 15% (w/v) at 200 rpm for 4 h at RT until achieving homogeneity, and afterwards, gelatine 15% (w/v) was slowly added, under agitation (Fig. 9B). This solution was left stirring overnight at 200 rpm at RT. Afterwards, 5% glucose was added to zein and gelatine solution and left stirring overnight at 200 rpm (Fig. 9C). This solution was denoted as zn.gel.glu. When *Nannochloropsis oceanica* (NO) powder was added at 10% w/v the solutions were denoted as zn.gel.glu.NO (Fig. 9D).

### Fabrication of zein-based electrospun nanofibres

The electrospun fibres were fabricated using a homemade electrospinning apparatus. The solutions were loaded into syringes and placed into a mechanical syringe pump. An applied voltage of 25 kV and a controlled flow rate of 1 mL/h were used to generate the zn.gel.glu fibres and the zn.gel.glu.NO fibres, creating an electrical potential between a 20 G stainless steel needle and a grounded static collector at 10 cm from the needle tip, comprised of either (i) a flat copper plate cover to aluminium foil perpendicular to the needle, when fabricating randomly orientated fibres; or (ii) two parallel copper plates (separated by 1.8 cm from each other) and placed on the same direction of the needle, when fabricating electrospun meshes termed as aligned fibres (AL). The temperature and relative humidity were monitored and controlled during electrospinning: the parameters varied between 20 °C and 23 °C and between 25% and 45%, respectively.

### Electrospun fibres crosslinking

After electrospinning, the zn.gel.glu fibres and the z.gel.glu.NO fibres were submitted to a thermal treatment at 140 °C for 3 h in a convection oven, as previously described^32^. After that, the fibres were cooled down at room temperature. This crosslinking by thermal treatment, also designated as the Maillard reaction, allows the conjugation of the glucose carbonyl groups and the free amino groups of gelatine, improving the mechanical strength of the fibres. These fibres were termed zn.gel.glu crosslinked and zn.gel.glu.NO crosslinked fibres.

### Scanning electron microscopy (SEM) analysis

The structural characterization of zein-based electrospun nanofibres was carried out by a field emission gun SEM (FEG-SEM) (Model JSM-7001F; JEOL, Tokyo, Japan). Before imaging, samples were mounted on a holder with carbon tape and coated with a 30 nm gold/palladium (60:40) layer (Model E5100 Sputter Coater; Polaron: Quorum Technologies, Lewes, UK). The samples were scanned at various magnifications with an average accelerating voltage of 15 kV. The average fibre diameter was estimated using the ImageJ software (National Institutes of Health, NIH, USA). The average fibre diameter for each condition was determined by measuring 100 individual nanofibres using four independent SEM images (*N* = 4, *n* = 100).

### Attenuated total reflection Fourier-transform infrared (ATR-FTIR) spectroscopy

The ATR-FTIR analysis was performed using a Spectrum Two FT-IR Spectrometer from PerkinElmer (Waltham, Massachusetts, U.S.A.), equipped with an attenuated total reflection (ATR) UATR Two accessory. ATR-FTIR spectra were collected to identify functional groups commonly found in zein and gelatine, and to evaluate the impact of the thermal treatment on the nanofibres. Transmittance spectra were obtained at room temperature over the spectral region from 400 cm^−1^ to 4000 cm^−1^, with a resolution of 4 cm^−1^ and eight scans of data accumulation. An automatic baseline correction treatment was applied using the acquisition software. All ATR-FTIR spectra were normalized using the maximum and minimum transmittance of each spectrum.

### Contact angle measurements

The contact angles of the zn.gel.glu fibres and the zn.gel.glu.NO fibres (with and without crosslinking treatment) were measured using a DSA25B Drop Shape Analyzer (Kruss, Hamburg, Germany) and the sessile drop method. Droplets of glycerol were placed on the surface of the various fibrous scaffolds and the contact angles were measured. A 3 s^−1^ rate of acquisition was used. For each condition, the contact angles were measured in at least 4 independent fibre mats (*n* = 4).

### Evaluation of mechanical properties

The mechanical properties of the zn.gel.glu fibres with and without the thermal treatment were tested under uniaxial tensile testing at room temperature using a mechanical tester (Model UV-200-01; CellScale Biomaterials Testing, Waterloo, Canada) with a 50 N load cell and a displacement rate of 3 mm min^−1^. First, the specimens from each experimental group (*n* = 5) were cut into rectangular strips and their thickness, width and length were measured. Experimental data was collected and processed using the UniVert software. The stress–strain curves were plotted using each sample cross-section area and initial lengths. With the stress–strain curves, the Young’s modulus (MPa) of the fibres was computed from the 0–15% initial linear region slope, while the ultimate tensile strength (UTS) (MPa) of each sample was obtained from the highest peak of each curve. Ultimate elongation (%) was determined by dividing the displacement measured at the highest peak of the stress–strain curve by the original length of each specimen.

### DLEC line culture

An embryonic-like fish cell line derived from European seabass (*Dicentrarchus labrax* Embryonic Cell Line, DLEC) was purchased from Kerafast (Boston, Massachusetts, USA) and cultured as previously reported^33^. Culture media was prepared using Leibovitz 15 medium (L-15 medium, Sigma Aldrich) with 10% (v/v) Foetal Bovine Serum (FBS), 1% (v/v) L-glutamine (Sigma Aldrich), 1% antibiotic–antimycotic (anti-anti) and 5 µL mL^−1^ of 3 M NaCl (L15 complete medium). DLEC cells were incubated at 25 °C without CO_2_. Media was exchanged every 2–3 days and passaging was done every 4–5 days, when confluency was reached, using 0.05% trypsin/ethylenediaminetetraacetic acid (EDTA) for 8 min at RT for cell detachment. The DLEC cells were recovered by centrifugation at 160 × *g* for 4 min. After carefully removing the supernatant, the cell pellet was resuspended in L-15 complete media. Cells were seeded at a density of 10,000 cells/cm^2^ into new plates containing complete culture media.

### Cell seeding and culture on the electrospun nanofibres

After electrospinning and post-processing thermal treatment, the electrospun fibres were fixed on glass coverslips (13 mm of diameter; VWR) with FDA-approved biocompatible adhesive silicone glue (Silastic Medical Adhesive Silicone, Type A; Dow Corning). The fibres were left overnight to glue accurately at RT. Afterwards, UV sterilization was performed for 5 h, followed by incubation with 2% anti-anti solution for 2 days at 25 °C. Then, the fibrous scaffolds were placed in ultra-low cell attachment 24-well plates (Corning). The scaffolds were then washed three times with PBS and incubated in culture medium overnight at 25 °C.

DLEC cells were seeded in the electrospun fibres at a density of 50,000 cells per scaffold. The cell-seeded scaffolds were incubated at 25 °C without CO_2_ for 2 h without culture medium to promote initial cell adhesion. The culture was conducted for 20 days, and culture media was changed every 2–3 days.

### Cell proliferation assay

The proliferation of DLEC cells on the zein-based electrospun fibres was evaluated using the AlamarBlue assay (AlamarBlue Cell Viability Reagent; Thermo Fisher Scientific) following the manufacturer’s guidelines. The metabolic activity of the cells seeded on the fibrous scaffolds and respective controls was monitored and estimated at days 3, 8, 14 and 20 of culture. Briefly, a 10% (v/v) AlamarBlue solution diluted in cell culture media was added to the scaffolds and incubated at 25 °C for 4 h. Fluorescence intensity was measured in a plate reader (Infinite 200 Pro; Tecan, Switzerland) at an excitation/emission wavelength of 560/590 nm. For each experimental group, the fluorescence intensity was analysed for five independent scaffolds (*n* = 5) and acellular electrospun scaffolds were used as blank controls. Fluorescence intensity values were measured in triplicate for each sample. The number of viable cells in each scaffold at each time point was estimated through a calibration curve that correlates different DLEC cell numbers counted with the respective obtained AlamarBlue assay fluorescence intensity.

### Fluorescence staining analysis

Fluorescence staining of DLEC cells cultured for 20 days in the electrospun fibres was performed by fixing cells in 4% paraformaldehyde (PFA, Sigma) in PBS for 20 min at RT. Nucleus staining was performed using DAPI (1.5 μg/mL in PBS) in PBS for 10 min at RT. Alexa Fluor 488® Phalloidin and Alexa Fluor 555® Phalloidin (Thermo Fisher Scientific) at a dilution factor of 1:150 in PBS were applied for 30 min at RT. A final washing step in PBS was performed before imaging in a Leica DMI3000B microscope (Leica Microsystems) and a LSM980 with Airyscan 2 confocal microscope (Zeiss).

### Scanning electron microscopy (SEM) analysis

SEM imaging was performed to allow the evaluation of the cell morphology of the cells cultured on the zein-based electrospun scaffolds for 20 days. Previously fixed samples in 4% PFA were first immersed in ethanol solutions with increasing concentrations (33.3%, 66.7%, 100% (v/v)), for 20 min each. After, the samples were also immersed in hexamethyldisilane (HMDS) and ethanol mixtures with increasing HMDS concentrations (33.3%, 66.7%, 100% (v/v)), for 20 min each, and left to dry overnight in a fume hood. Before SEM imaging, the scaffolds were sputter-coated with a gold/palladium layer, as previously described.

### Prototype fabrication

Two different moulds were 3D printed by fused deposition modelling (FDM) using a Prusa i3 MK3S 3D extruder (PRUSA Research, Prague, Czech Republic) loaded with a PLA filament: one consisting of a 50 mm × 41 mm box, and other consisting on an insert with 49 mm × 40 mm and six diagonal plates that recapitulate the myoseptum. During the casting process, the moulds were filled with a 0.9% w/v κ-carrageenan hydrogel supplemented with 1% w/v beetroot powder at room temperature until gelation was completed. Subsequently, the moulds were carefully removed and zn.gel.glu.NO crosslinked fibres (AL) were included to mimic the myoseptum. The analysis of the interface between these two scaffolds was carried out by SEM (FEG-SEM). For that, a sample was dried by sequential submerging in ethanol solutions of increased concentrations varying from 10% (v/v) to 97.8% (v/v). Samples were immersed for 30 min in each ethanol concentration. After that, samples were immersed in hexamethyldisilane (HDMS) for 1 h and air-dried overnight.

### Statistical analysis

Data is presented as mean values ± standard deviation. A Shapiro–Wilk test was used for assessing the normality distribution of the samples. Statistical differences were calculated using either the unpaired Student’s *t* test (two-groups data sets), ordinary one-way ANOVA, or two-way ANOVA. Statistical significance (*p < 0.05, **p < 0.01, ***p < 0.001, ****p < 0.0001) was evaluated by Post-hoc Tukey’s multiple comparison test or by Post-hoc Šidák’s multiple comparison test. All analysis were preformed using GraphPad Prism 10.2.1 (GraphPad, San Diego, CA, USA).

## Supplementary information

SI_zn.gel.glu.NO fibres_vfinal

## Data Availability

The data generated from this work is available and can be provided upon request to the corresponding authors.
