# Supplementary material for: Mimicking the myoseptum in cultivated fish by manufacturing edible microalgae-rich nanofibers
Source: NPJ Sci Food. 2025 Jul 21;9:145. doi: 10.1038/s41538-025-00508-6 (PMC12279974; doi:10.1038/s41538-025-00508-6)
Supplement: Supplementary file 1 — SI_zn.gel.glu.NO fibres_vfinal [file 41538_2025_508_MOESM1_ESM.pdf]

SUPPORTING INFORMATION

Mimicking the myoseptum in cultivated fish by manufacturing edible microalgae-rich nanofibers

Diana M. C. Marques[1,2], Bernardo Pereira[1], Beatriz Malhão[1], João C. Silva[1,2,3], Paola Sanjuan-Alberte[1,2], Frederico Castelo Ferreira[1,2]*

[1]Department of Bioengineering and Institute for Bioengineering and Biosciences, Instituto Superior Técnico, Universidade de Lisboa, Av. Rovisco Pais, 1049-001 Lisbon, Portugal

[2]Associate Laboratory i4HB—Institute for Health and Bioeconomy, Instituto Superior Técnico, Universidade de Lisboa, Av. Rovisco Pais, 1049-001 Lisbon, Portugal

[3]Department of Mechanical and Aerospace Engineering and PolitoBIOMed Lab, Politecnico di Torino, Corso Duca Degli Abruzzi, 24, Turin, 10129 Italy

*Corresponding authors: frederico.ferreira@tecnico.ulisboa.pt

Table S1. Electrospun scaffolds components and respective nomenclature.

Nomenclature	Component
zn	Zein
gel	Gelatine
glu	Glucose
NO	*N. oceanica*

Table S2. Electrospun scaffolds nomenclature and quantity of each component.

Sample	Zein (mg)	Gelatine (mg)	Glucose (mg)	*N. oceanica* (mg)	Ethanol (mL)	Acetic acid (mL)
30% zein/ethanol	3	-	-	-	10	-
40% zein/ethanol	4	-	-	-	10	-
30% zein/acetic acid	3	-	-	-	-	10
40% zein/acetic acid	4	-	-	-	-	10
zn.gel.glu fibres	1.5	1.5	0.5	-	-	10
zn.gel.glu crosslinked fibres	1.5	1.5	0.5	-	-	10
zn.gel.glu.NO fibres	1.5	1.5	0.5	1	-	10
zn.gel.glu.NO crosslinked fibres	1.5	1.5	0.5	1	-	10

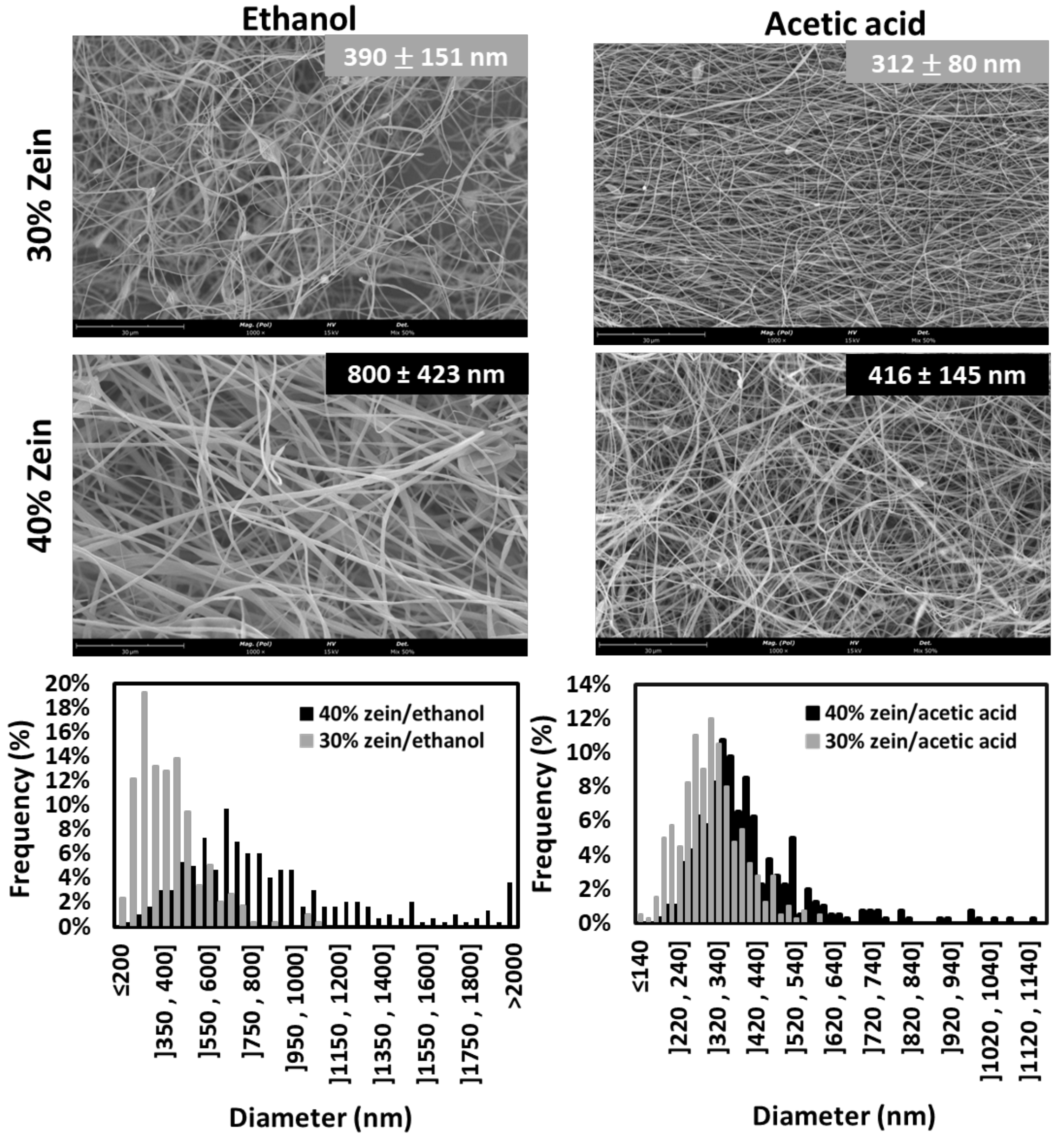

Figure S1. SEM micrographs of 30% zein electrospun fibres fabricated using 70% ethanol (A) and (B) 80% acetic acid and 40% zein fibres fabricated using (C) 70% ethanol and (D) 80% acetic acid. Fibre diameter histograms of different zein electrospun scaffolds fabricated using ethanol (E) and acetic acid (F). Scale bar: 20 µm.

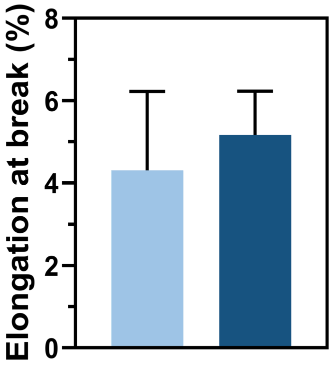

Figure S2. Elongation at break of the zn.gel.glu and zn.gel.glu.NO fibrous scaffolds fabricated with and without crosslinking obtained after tensile testing. Four independent sample specimens (n=4) were used in the analysis. Statistical significance was assessed using the unpaired t-student test.

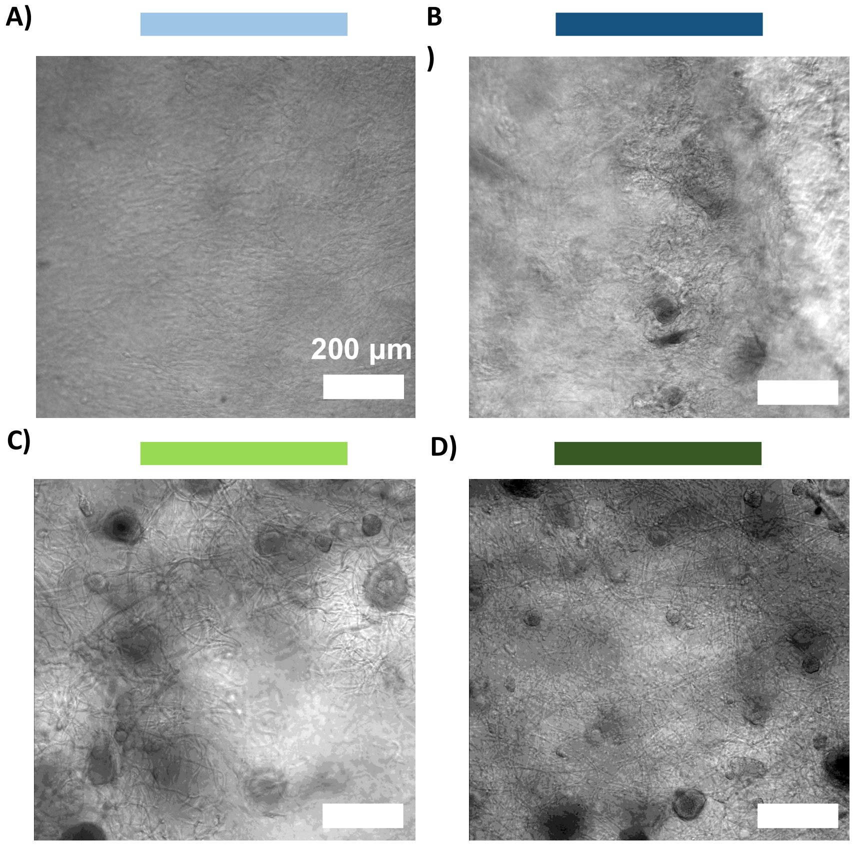

Figure S3. Brightfield confocal microscopy images of DLEC cells cultured on A) zn.gel.glu fibres, B) zn.gel.glu crosslinked fibres, C) zn.gel.glu.NO fibres, and D) zn.gel.glu.NO crosslinked fibres, after 20 days of culture. Scale bar: 200 µm.

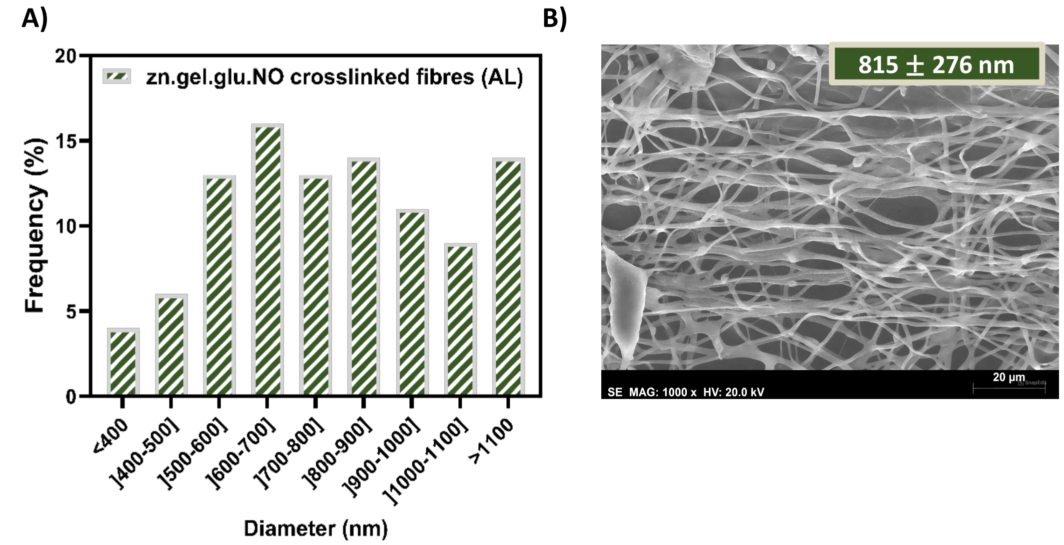

Figure S4. **A)** Fibre diameter histogram of the zein.gel.glu.NO crosslinked fibres (AL) and respective **B)** SEM micrograph. Scale bar: 20 µm.

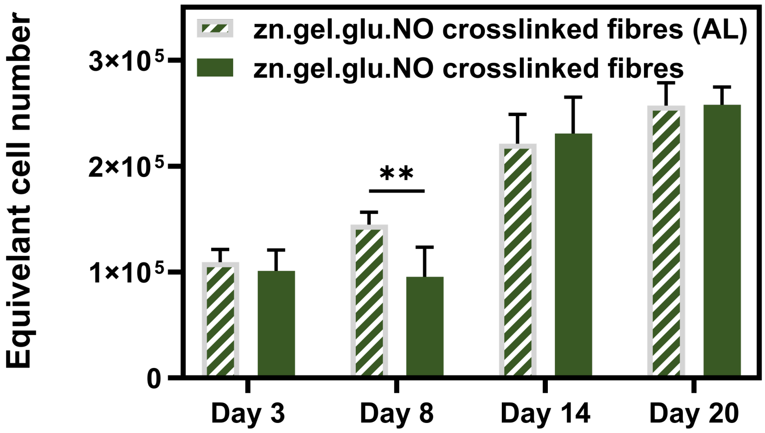

Figure S5. AlamarBlue cell metabolic activity assay of DLEC cultured on zn.gel.glu.NO crosslinked fibres (AL) and zn.gel.glu.NO crosslinked fibres. Five different samples (n=5) were used for the analysis. Statistical significance was assessed with different significance values (*$p < 0.05$, **$p < 0.01$, ***$p < 0.001$, ****$p < 0.0001$) using ordinary two-way ANOVA and Šidák's multiple comparison test (** = 0.0087).

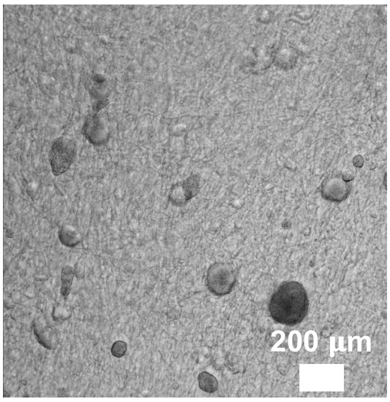

Figure S6. Brightfield confocal microscopy images of DLEC cells cultured on zn.gel.glu.NO crosslinked fibres (AL), after 20 days of culture. Scale bar: 200 µm.

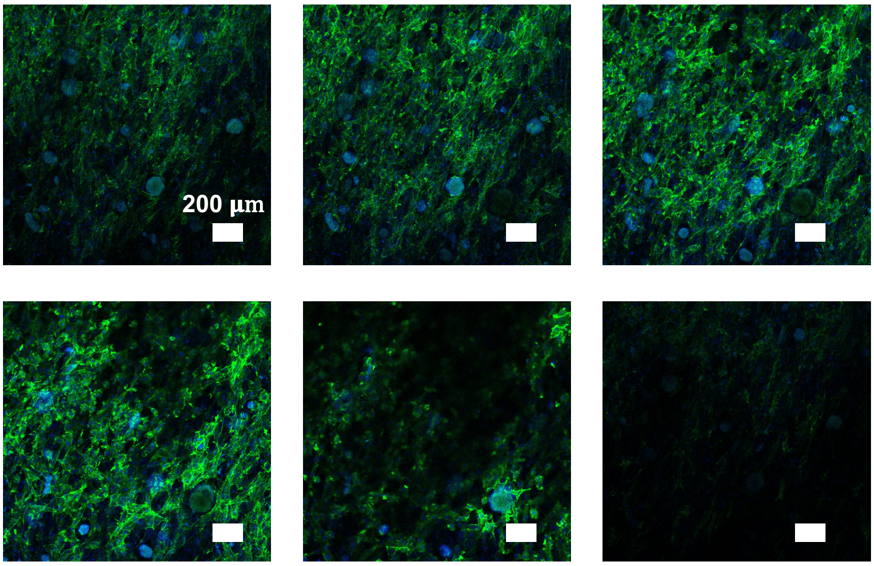

Figure S7. Confocal microscopy z-stack image of DAPI/Phalloidin staining using green phalloidin staining for DLEC cytoskeleton and blue DAPI staining for DLEC nuclei. Staining of DLEC cells cultured on zn.gel.glu.NO crosslinked fibres (AL), after 20 days of culture. From top left to bottom right is possible to observe cell migration throughout different z-stacks (from Z1 to Z41). Scale bar: 200 µm.
